# 

**Published:** 1900-03

**Authors:** 


Secretary.

---

### PERSONALS.

Dr. A. D. Galbraith, of Greensburg, Ind., is now located at Knoxville, Tenn.

Mr. Alexander Eger, the veterinary bookseller and publisher of Chicago, made a visit to all the leading veterinary colleges and centres in February.

Dr. G. E. Hutton, of Kansas, has been selected superintendent of the Monmouth (Ill.) driving-park.

Dr. E. P. Niles, of Blacksburg, Va., recently purchased a very speedy trotting mare, "Nelly Jewett."

Dr. Andrew Smith, of Toronto, is one of the directors of the Canadian Hackney Society.

Dr. H. D. Gill, of the JOURNAL staff, added another fast trotter to his string at the Fasig-Tipton sale in New York in January.

Dr. F. W. Huntington, of Portland, Maine, has retired as secretary and manager of Rigby Park Track Association.

Dr. Stephen L. Blount, who recently accompanied a consignment of mules to South Africa for the English Government, has returned again to the United States. Out of some nine hundred animals shipped less than thirty were lost from the various diseases and accidents common to ocean voyages. Dr. Blount met with a painful accident himself in falling down a hatchway, but fortunately no serious injuries were the result of the mishap.

Dr. J. R. Hagyard, of Lexington, Ky., has associated with him in his practice and infirmary Dr. James T. Shannon.

Farmer Miles, of Charleston, Ill., exacts of his pupils a royalty of five dollars for each ridgling castrated for a period of ten years after being instructed.

Dr. Fred. Evans, of Grand Island, Neb., is the owner of a fast-stepping colt by the noted sire "Guelph." This promising colt and the enjoyment of an increasing practice makes Dr. Evans' lot in life a happy one.

Dr. W. H. Heckenberger, of Catasauqua, Pa., recently met with a painful accident, injuring his hip and confining him to his bed.

Dr. A. Hanhoeffer, of Strasburg, Ont., has succeeded Dr. Owen Reist as sanitary inspector for his district.

Dr. Owen Reist, of Kossuth, Ont., resigned the position of sanitary inspector of Waterloo township and entered the race for municipal councillor, to which he was elected.

Dr. Frank T. Shannon, of the Bureau of Animal Industry, has been transferred from Los Angeles, Cal., to Washington, D. C.

Drs. Leonard Pearson, William H. Ridge, and C. J. Marshall have been selected to fill the rôle of veterinarians to the Philadelphia horse-show.

Dr. M. Jacobs, resident surgeon of the veterinary department, and Messrs. Cornman and Young, of the senior class of the University of Pennsylvania, were visitors to the dog-show in New York in February.

Dr. John J. Repp, of Ames, Iowa, reports a hopeful outlook for veterinary legislation in that State. The proposed law regulating the practice of veterinary science was favorably considered by the lower branch of the legislature.

Dr. J. Stewart Lacock, of Allegheny, was a visitor to Philadelphia in February on his return from the New York dog-show.

Dr. Alexander Glass, of Philadelphia, was an interested spectator of the many valuable canines shown in New York at the national dog-show.

Dr. John Wende, of Buffalo, N. Y., has just suffered the unexpected loss of his brother, Theodore Wende, a prominent member of the Buffalo Bar. Death followed an operation for appendicitis of a few days' development.

Dr. Thomas B. Young, of Media, Pa., fills the rôle of Recorder for Delaware County, and takes an active part in political affairs in that county.

Dr. J. S. Pollard, of Providence, R. I., fills the rôle of State veterinarian for that Commonwealth.

Dr. William Dougherty, of Baltimore, Md., has been on the sick-list for some two weeks. Dr. Martinet has been taking care of his practice.

Among the boardwalk visitors to Atlantic City on Sunday, March 11th, were to be noted Dr. A. T. Sellers, of Camden, N. J., and Dr. W. Horace Hoskins, of Philadelphia, both of whom have now large commercial interests in this Mecca by the sea.

Dr. Bryan Pendry has passed the necessary examination and received a promotion to second lieutenant of the Thirteenth Regiment of Greater New York.

Dr. William Folsetter, of Dallas, Tex., and Dr. W. A. Knight, of Houston, Tex., pursued a post-graduate course at the Kansas City Veterinary College during January and February.

Dr. Leonard Pearson, of Philadelphia, was a visitor and speaker at several meetings of Farmers' Institutes in Western Pennsylvania in March.

The Kansas City Kennel Club held its third annual bench show in the Kansas City convention hall. The display of dogs was excellent, the attendance was large, and the show was a howling success in every particular. Dr. R. C. Moore served as official veterinarian.

